# High Risk for Invasive Meningococcal Disease Among Patients Receiving Eculizumab (Soliris) Despite Receipt of Meningococcal Vaccine

**DOI:** 10.15585/mmwr.mm6627e1

**Published:** 2017-07-14

**Authors:** Lucy A. McNamara, Nadav Topaz, Xin Wang, Susan Hariri, LeAnne Fox, Jessica R. MacNeil

**Affiliations:** 1 Division of Bacterial Diseases, National Center for Immunization and Respiratory Diseases, CDC.

Use of eculizumab (Soliris, Alexion Pharmaceuticals), a terminal complement inhibitor, is associated with a 1,000-fold to 2,000-fold increased incidence of meningococcal disease (*1*). Administration of meningococcal vaccines is recommended for patients receiving eculizumab before beginning treatment (*2*,*3*). Sixteen cases of meningococcal disease were identified in eculizumab recipients in the United States during 2008–2016; among these, 11 were caused by nongroupable *Neisseria meningitidis*. Fourteen patients had documentation of receipt of at least 1 dose of meningococcal vaccine before disease onset. Because eculizumab recipients remain at risk for meningococcal disease even after receipt of meningococcal vaccines, some health care providers in the United States as well as public health agencies in other countries recommend antimicrobial prophylaxis for the duration of eculizumab treatment; a lifelong course of treatment is expected for many patients. Heightened awareness, early care seeking, and rapid treatment of any symptoms consistent with meningococcal disease are essential for all patients receiving eculizumab treatment, regardless of meningococcal vaccination or antimicrobial prophylaxis status.

Eculizumab is licensed in the United States for treatment of paroxysmal nocturnal hemoglobinuria and atypical hemolytic uremic syndrome (*2*); both are rare, life-threatening illnesses. The Food and Drug Administration (FDA)–approved prescribing information includes a boxed warning regarding increased risk for meningococcal disease in eculizumab recipients (*2*). To mitigate the occurrence of and morbidity associated with meningococcal infections, FDA requires a Risk Evaluation and Mitigation Strategy (REMS) (http://www.solirisrems.com/) to educate health care providers and patients about the risk for and early signs of possible meningococcal infection and the need for immediate medical evaluation of signs and symptoms consistent with possible meningococcal infection. A key element of the Soliris REMS is ensuring that patients receive meningococcal vaccines.* The Advisory Committee on Immunization Practices recommends that eculizumab recipients receive both quadrivalent meningococcal conjugate (MenACWY) and serogroup B (MenB) meningococcal vaccines (*3*).

In February 2017, CDC requested that health departments review existing meningococcal disease case investigation records since 2007 to identify cases in eculizumab recipients; isolates or clinical specimens for identified cases were also requested for additional characterization. The requests were made through Epi-X (https://www.cdc.gov/epix/), CDC’s secure communications network for public health officials, and follow-up with each health department occurred through individual e-mail correspondence. Forty-seven state health departments and the health departments of New York City and the District of Columbia responded to CDC’s request for information. A search of the FDA Adverse Events Reporting System identified additional information on meningococcal vaccines received by patients identified through the Epi-X request.

CDC’s Bacterial Meningitis Laboratory performed slide agglutination,^†^ polymerase chain reaction (PCR) testing, and whole genome sequencing (WGS) on isolates to determine the serogroup (*4*); the serogroup for one clinical specimen with no isolate was determined by PCR. The serogroup results from slide agglutination (nongroupable) and WGS (serogroup C) differed for one isolate. For that isolate, the slide agglutination result (nongroupable) was used in analysis, because slide agglutination detects expression of the polysaccharide capsule, which is necessary for protection by MenACWY vaccines. Antimicrobial susceptibility testing also was performed.

In response to the Epi-X request, 16 meningococcal disease cases in eculizumab recipients were identified for the period 2008–2016 from 10 jurisdictions. The median patient age was 30 years (range = 16–83 years). All patients had meningococcemia; six also had evidence of meningitis. Patients were hospitalized for an average of 6.6 days (range = 1–14 days); one patient died (case-fatality ratio = 6%). Ten of the 16 patients were receiving eculizumab for paroxysmal nocturnal hemoglobinuria, five for atypical hemolytic uremic syndrome, and one for another condition, through a clinical trial.

Isolates from 14 patients were available for further characterization; a clinical specimen, but no isolate, was available for one patient; and for one patient no clinical specimen or isolate was available. Four cases were determined to be caused by serogroup Y and 11 by nongroupable *N. meningitidis* (Table 1). Antimicrobial susceptibility testing was performed on the 14 isolates (Table 2). One patient infected with a penicillin intermediate-susceptible strain had been prescribed penicillin prophylaxis, although the patient reported poor compliance. Further characterization of these isolates is ongoing.

**TABLE 1:** Meningococcal vaccination status and disease-causing serogroup in eculizumab recipients with meningococcal disease (N = 16) — 10 U.S. jurisdictions, 2008–2016

Characteristic	No. (%)
MenACWY vaccination*	
Yes	14 (88)
No/unknown	2 (12)
**MenB vaccination (patients with diagnosis after June 12, 2015)^†^**	
Yes^§^	3 (75)
No/unknown	1 (25)
**Disease-causing serogroup**	
B	0 (—)
C	0 (—)
Y	4 (25)
Nongroupable^¶^	11 (69)
Not determined	1 (6)

* MenACWY vaccination includes MenACWY conjugate vaccine, meningococcal polysaccharide vaccine, and meningococcal vaccine of unknown type. Only vaccines received before disease onset are included.

^†^ MenB vaccines were licensed for use in the United States in 2014 and 2015. Advisory Committee on Immunization Practices recommendations for use of MenB vaccine in persons at increased risk for serogroup B meningococcal disease were published on June 12, 2015. No patients had received MenB-FHbp (Trumenba, Pfizer Vaccines); three patients had received MenB-4C (Bexsero, GlaxoSmithKline). Only vaccines received before disease onset are included.

^§^ Includes 1 or 2 doses of MenB vaccine.

^¶^ Includes one patient for whom no isolate was available but classified as nongroupable based on polymerase chain reaction testing on a clinical specimen.

**TABLE 2:** Antimicrobial susceptibility testing on isolates from eculizumab recipients (N = 14) with meningococcal disease — 10 U.S. jurisdictions, 2008–2016

Antibiotic	Susceptibility (No.)			
	Susceptible	Intermediate	Resistant	Nonsusceptible*
Ampicillin	11	3	0	N/A
Ceftriaxone	14	0	0	N/A
Ciprofloxacin	13	0	1	N/A
Penicillin	10	3	1	N/A
Rifampin	14	0	0	N/A
Trimethoprim-sulfamethoxazole	2	1	11	N/A
Azithromycin	14	N/A	N/A	0

**Abbreviation:** N/A = not applicable.

* Breakpoints for intermediate susceptibility versus resistance not established.

Fourteen patients had documentation of receipt of MenACWY before disease onset (Table 1). Three of four meningococcal disease cases diagnosed after publication of the ACIP recommendations for use of MenB vaccine in persons at increased risk occurred in patients with documentation of receipt of 1 or more doses of MenB vaccine before disease onset. Three of four patients with serogroup Y disease had documentation of previous MenACWY receipt.

## Discussion

Meningococcal disease following MenACWY vaccination in eculizumab recipients has been reported previously (*1*,*5*), and in vitro data have shown that eculizumab impairs meningococcal killing in whole blood even in subjects vaccinated against the relevant meningococcal serogroup (*6*). In addition, although nongroupable *N. meningitidis* is often carried asymptomatically in the nasopharynx, it rarely causes disease in healthy persons (*7*).

MenACWY vaccines target the serogroup-specific polysaccharide capsule and provide no protection against nongroupable *N. meningitidis*. MenB vaccines are licensed specifically for protection against serogroup B meningococcal disease. The extent of any potential cross-protection has not been assessed. The evidence of meningococcal disease in eculizumab recipients vaccinated against the infecting serogroup, together with the susceptibility of these persons to nongroupable meningococcal strains, is consistent with the in vitro data and suggests that eculizumab therapy interferes with the ability of antimeningococcal antibodies to provide protection against invasive disease.

Many clinicians and public health agencies, particularly in the United Kingdom and France, recommend antimicrobial prophylaxis with penicillin for the duration of eculizumab treatment; macrolides are typically recommended for penicillin-allergic patients (*8*).^§^ Long-term penicillin prophylaxis is generally considered to be safe,^¶^ although the effectiveness of this strategy for meningococcal disease prevention has not been established. Ten of the 14 isolates characterized in this analysis were fully susceptible to penicillin, three demonstrated intermediate penicillin susceptibility, and one was resistant to penicillin. This finding is consistent with recent studies of invasive meningococcal isolates in the United States, which have shown that most isolates are fully susceptible to penicillin and that penicillin resistance is very rare (*9*). The clinical implications of intermediate penicillin susceptibility are unclear. Meningococcal disease caused by both penicillin-resistant *N. meningitidis* and *N. meningitidis* with intermediate penicillin susceptibility have been reported in eculizumab recipients taking penicillin or amoxicillin prophylaxis (*7*,*10*), but patient compliance was not reported.

Although neither meningococcal vaccination nor antimicrobial prophylaxis can be expected to prevent all cases of meningococcal disease in eculizumab recipients, providers should continue to follow ACIP recommendations for eculizumab recipients to receive both MenACWY and MenB vaccines. Providers could also consider antimicrobial prophylaxis for the duration of eculizumab treatment to potentially reduce the risk for meningococcal disease. Data will continue to be evaluated and additional guidance will be developed as evidence becomes available. Heightened awareness and vigilance for symptoms consistent with meningococcal disease are essential for all patients receiving eculizumab treatment and their health care providers, regardless of meningococcal vaccination or antimicrobial prophylaxis status.

Of note, 10 cases in this report had meningococcemia without meningitis. Although a petechial or purpuric rash is a hallmark of meningococcemia, this rash might not appear until later stages of illness. Initial symptoms of meningococcemia are often relatively mild and nonspecific, and might include fever, chills, fatigue, vomiting, diarrhea, and aches or pains in the muscles, joints, chest, or abdomen; however, these symptoms can progress to severe illness and death within hours. Health care providers should have a high index of suspicion for meningococcal disease in patients taking eculizumab who develop any symptoms consistent with either meningitis or meningococcemia, even if the patient’s symptoms initially appear mild, and even if the patient has been fully vaccinated or is receiving antimicrobial prophylaxis.

State health departments are asked to complete a supplemental case report form (available at https://www.cdc.gov/meningococcal/surveillance/index.html) for all meningococcal disease cases occurring among eculizumab recipients; forms should be submitted to CDC via secure e-mail (meningnet@cdc.gov) or secure fax (404–471–8372), along with any available isolates for whole genome sequencing.

**Summary**

**What is already known about this topic?**

Eculizumab (Soliris, Alexion Pharmaceuticals), a terminal complement inhibitor, is associated with a 1,000-fold to 2,000-fold increased incidence of meningococcal disease among persons receiving the drug. The Food and Drug Administration (FDA)–approved prescribing information includes a boxed warning regarding increased risk for meningococcal disease in eculizumab recipients. The Advisory Committee on Immunization Practices recommends both MenACWY and MenB vaccination for patients taking eculizumab.

**What is added by this report?**

Following review of existing meningococcal disease case investigation records, 16 cases of meningococcal disease were identified in eculizumab recipients in the United States for the period 2008–2016. The majority of cases were caused by nongroupable *Neisseria meningitidis* and occurred in patients who had documentation of receipt of at least 1 dose of meningococcal vaccine before disease onset.

**What are the implications for public health practice?**

Health care providers should continue to follow recommendations from the Advisory Committee on Immunization Practices for eculizumab recipients to receive both MenACWY and MenB vaccines and could consider antimicrobial prophylaxis for the duration of eculizumab treatment to potentially reduce the risk for meningococcal disease. However, neither vaccination nor antimicrobial prophylaxis can be expected to prevent all cases of meningococcal disease in eculizumab recipients. Heightened awareness, early care seeking, and rapid treatment of any symptoms consistent with meningococcal disease are essential in all patients receiving eculizumab treatment, regardless of meningococcal vaccination or antimicrobial prophylaxis status.

## Footnotes

* FDA background package for meeting of drug safety and risk management advisory Committee. https://www.fda.gov/downloads/AdvisoryCommittees/CommitteesMeetingMaterials/Drugs/DrugSafetyandRiskManagementAdvisoryCommittee/UCM423030.pdf.

† Laboratory methods for the diagnosis of meningitis caused by *Neisseria meningitidis*, *Streptococcus pneumoniae*, and *Haemophilus influenzae*. https://www.cdc.gov/meningitis/lab-manual/full-manual.pdf.

§ PNH National Service Leeds. 2017. Meningococcal infection and eculizumab. http://www.pnhleeds.co.uk/professionals/meningococcal-infection-and-eculizumab/.

¶ Safety of long term therapy with penicillin and penicillin derivatives. https://www.fda.gov/drugs/emergencypreparedness/bioterrorismanddrugpreparedness/ucm072755.htm.
